# Advances in pharmacological effects and mechanism of action of cinnamaldehyde

**DOI:** 10.3389/fphar.2024.1365949

**Published:** 2024-06-06

**Authors:** Jiageng Guo, Shidu Yan, Xinya Jiang, Zixia Su, Fan Zhang, Jinling Xie, Erwei Hao, Chun Yao

**Affiliations:** ^1^ Guangxi Key Laboratory of Efficacy Study on Chinese Materia Medica, Guangxi University of Chinese Medicine, Nanning, China; ^2^ Guangxi Collaborative Innovation Center of Study on Functional Ingredients of Agricultural Residues, Guangxi University of Chinese Medicine, Nanning, China; ^3^ Guangxi Key Laboratory of TCM Formulas Theory and Transformation for Damp Diseases, Guangxi University of Chinese Medicine, Nanning, China; ^4^ Ruikang Hospital Affiliated to Guangxi University of Chinese Medicine, Nanning, China; ^5^ Engineering Research Center of Innovative Drugs for Traditional Chinese Medicine and Zhuang and Yao Medicine, Ministry of Education, Guangxi University of Chinese Medicine, Nanning, China

**Keywords:** cinnamaldehyde, pharmacological action, mechanism of action, research progress, traditional Chinese medicine

## Abstract

Cinnamaldehyde is extracted from *Cinnamomum cassia* and other species, providing diverse sources for varying chemical properties and therapeutic effects. Besides natural extraction, synthetic production and biotechnological methods like microbial fermentation offer scalable and sustainable alternatives. Cinnamaldehyd demonstrates a broad pharmacological range, impacting various diseases through detailed mechanisms. This review aims to encapsulate the diverse therapeutic effects of cinnamaldehyde, its molecular interactions, and its potential in clinical applications. Drawing on recent scientific studies and databases like Web of Science, PubMed, and ScienceDirect, this review outlines cinnamaldehyde’s efficacy in treating inflammatory conditions, bacterial infections, cancer, diabetes, and cardiovascular and kidney diseases. It primarily operates by inhibiting the NF-κB pathway and modulating pro-inflammatory mediators, alongside disrupting bacterial cells and inducing apoptosis in cancer cells. The compound enhances metabolic health by improving glucose uptake and insulin sensitivity and offers cardiovascular protection through its anti-inflammatory and lipid-lowering effects. Additionally, it promotes autophagy in kidney disease management. Preclinical and clinical research supports its therapeutic potential, underscoring the need for further investigation into its mechanisms and safety to develop new drugs based on cinnamaldehyde.

## 1 Introduction

Cinnamon, a dried bark originating from *Cinnamomum* genus, also referred to as fungus cinnamon and peony cinnamon, primarily originates from Guangxi, Guangdong, and Hainan provinces in China (Wang et al., 2023). Cinnamon has been utilized for centuries as both an herbal remedy and a culinary spice. (Li et al., 2023). In the realm of traditional medicine, cinnamon aids in the regulation of the body’s Yang energy by limiting inflammation, promoting the return of energy to its source, dispersing coldness, alleviating discomfort, and promoting the flow of energy in the body’s meridians. Moreover, this versatile ingredient is employed to address numerous conditions, such as erectile dysfunction and uterine coldness, cold pain in the lower back and knees, kidney deficiency and respiratory problems, Yang deficiency, dizziness and eye redness, cold discomfort in the heart and abdomen, cold-related diarrhea, hernia, abdominal pain, painful menstruation, etc. (HouXiaotao et al., 2018)Scientific studies have demonstrated the diverse pharmacological impacts of cinnamon, including its abilities to mitigate inflammation, combat bacterial infections, lower blood glucose levels, protect the heart, inhibit oxidative stress, decelerate the aging process, and even fight tumors (Sharifi-Rad et al., 2021).

**FIGURE 1:**
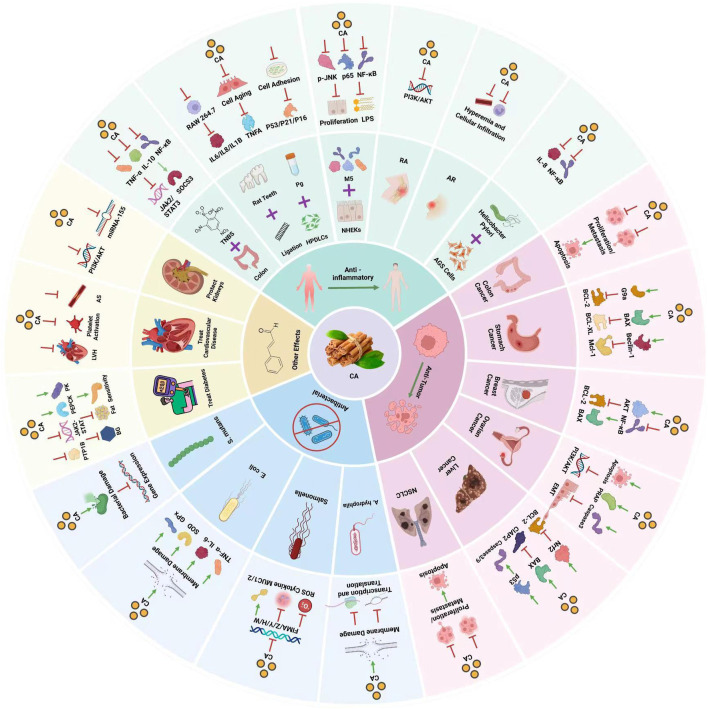
Pharmacological effects of cinnamaldehyde.

Furthermore, the anti-inflammatory and immunomodulatory characteristics of cinnamon are evident as they effectively suppress acute, subacute, and synchronic inflammatory reactions (Rathi, 2013). Research (Shan et al., 2007; Goyal et al., 2009)conducted on cinnamon and its constituents has indicated varying levels of effectiveness against a range of bacteria including *Staphylococcus aureus (S. aureus)*, *Escherichia coli* (*E. coli*), *Enterobacter aerogenes (E. aerogenes)*, *Aspergillus A.)*, *Pseudomonas aeruginosa (P. aeruginosa)*, *V. cholerae (Vibrio cholerae)*, *V. parahaemolyticus (Vibrio parahaemolyticus)*, and *Salmonella S.)*, filamentous fungi (e.g., *Pseudomonas* and *Candida albicans*), and three different types of dermatophytes (e.g., *Stachybotrys* and *Trichophyton rubrum*) (Ooi et al., 2006). Studies (Jamali et al., 2020) have further confirmed that cinnamon can lower the levels of blood sugar by improving those of glucose, triacylglycerol, low-density lipoprotein (LDL), and total cholesterol in patients with type 2 diabetes (Allen et al., 2013). In addition, it exhibits noteworthy cardioprotective properties by enhancing the supply of blood to the heart muscle, inhibiting the formation of blood clots in veins or arteries, and augmenting the flow of blood through the coronary arteries in isolated hearts. Additionally, it serves as a natural antioxidant within the contemporary food industry. Cinnamon consists of multiple chemical constituents, comprising volatile oil, flavonoids, variations of flavors, terpenoids, lignans, phenolic acids, coumarins, saponins, polysaccharides, and other elements (HouXiaotao et al., 2018). Furthermore, it encompasses inorganic components and alternative compounds (Manzoor et al., 2023). It should be highlighted that cinnamaldehyde emerges as the primary active constituent within cinnamon. Cinnamaldehyde, the primary active component in cinnamon, not only comes from *Cinnamomum cassia* but also other species such as *Cinnamomum zeylanicum* (Sadhna et al., 2022) and *Cinnamomum burmannii.* (Dimas et al., 2021).This broadens its source base and permits the exploration of various chemical properties and biological activities. Besides natural extraction, cinnamaldehyde can be synthetically produced through the condensation of benzaldehyde and acetaldehyde (Manash et al., 2020), and recent biotechnological advances have enabled its production via microbial fermentation, (Aleku et al., 2022), providing scalable and environmentally sustainable methods of obtaining this valuable compound. This article presents an overview of the presently accessible body of literature regarding the pharmacological impacts of cinnamaldehyde in cinnamon and discusses the mechanism underlying its effects. (Figure 1).

## 2 Review methodology

To comprehensively review the pharmacological effects of cinnamaldehyde, a systematic search was conducted across major scientific databases including Medline, PubMed, ScienceDirect, and Scopus, the range for the publication time is from 1st January 2013 to 31st November 2023. Moreover, a manual search was conducted to figure out pertinent articles. The literature retrieval process was designed to encompass a wide array of studies detailing both the molecular mechanisms and therapeutic potential of cinnamaldehyde. The search strategy employed the use of specific keywords: “Cinnamaldehyde” “mechanisms of action”, and “Pharmacological effects”, along with their relevant synonyms and related terms. The selection criteria for studies included in this review were predefined to include peer-reviewed research articles, review papers, and clinical trial reports published in English. Both *in vitro* and *in vivo* studies were considered to provide a holistic overview of cinnamaldehyde’s pharmacological profile. Exclusion criteria were set to omit articles not directly related to the pharmacological actions of cinnamaldehyde or those that focused solely on its chemical synthesis without addressing its biological effects.

## 3 Anti-inflammatory effect

### 3.1 Suppression of gastritis caused by *Helicobacter pylori (H. pylori)*

*Helicobacter pylori* is one of the prevailing chronic pathogenic bacteria. In Asian developing countries, this immensely contagious bacterium is significantly linked to tumor formation. The International Agency for Research on Cancer has classified *H. pylori* as a class 1 carcinogen for human gastric cancer (Duan et al., 2023). Currently, the management of *H. pylori* entails triple and quadruple therapy (Malfertheiner et al., 2023). However, this treatment is ineffective in a certain proportion of patients. The development of strong resistance by pathogenic bacteria greatly limits the use of anti-microbial drugs in clinical practice. Therefore, the identification of novel drugs for the treatment of *H. pylori* is an urgent clinical need.

Even though *H. pylori* is not invasive, it can induce a robust inflammatory and immune reaction. These effects enhance the synthesis of inflammatory cytokines, such as interleukin 1 (IL1), IL6, tumor necrosis factor-alpha (TNF-α), IL8, etc. Significantly, IL8 acts as a powerful chemokine that stimulates neutrophils and initiates the entry of acute inflammatory cells into the mucosa. (Crabtree et al., 1995).

Muhammad et al. (Muhammad et al., 2015). Studied the impact of cinnamaldehyde on *H. pylori*, as well as its cytotoxicity and ability to prevent adhesion. AGS/MKN-45 cells were co-cultured with *H. pylori*. Various techniques, including enzyme-linked immunosorbent assay, real-time polymerase chain reaction (PCR), and Western blotting, were employed to examine IL8 secretion and determine the effect of cinnamaldehyde on nuclear factor-kappa B (NF-κB) activation. This evaluation aimed to understand the anti-inflammatory mechanism of cinnamaldehyde on gastric epithelial cells infected with *H. pylori*. According to the experimental results, non-cytotoxic cinnamaldehyde does not have a bactericidal effect within a given time. This evidence indicated that the effect in the co-culture system of *H. pylori* and cells is not caused by the change in the activity of *H. pylori* to produce toxicity to cells. In addition, cinnamaldehyde can significantly inhibit the secretion and expression of IL8 in *H. pylori*-infected cells. Further studies have found that it can also inhibit the activation of NF-κB induced by *H. pylori* and the degradation of inhibitor of NF-κB (IκB).

In conclusion, cinnamaldehyde can offer benefits against gastritis caused by *H. pylori* by inhibiting the activation of NF-κB in AGS cells and downregulating the expression of IL8 induced by *H. pylori*.

### 3.2 Suppression of ulcerative colitis

Ulcerative colitis is a chronic inflammatory disease affecting the colon (Berre et al., 2023). The main feature of this disease is the presence of inflammation that is limited to the colon; it begins in the rectum, spreads continuously proximally, and often involves the peripatetic. The inflammatory changes in colitis are confined to the mucosa and submucosa, and associated with cryptitis, crypt abscesses, and infiltration of neutrophils, lymphocytes, natural killer cells, and monocytes (Liu and Stappenbeck, 2016). Symptoms of ulcerative colitis include abdominal cramps and diarrhea, which progress to weight loss, fatigue, rectal bleeding, fever, and anemia (Head and Jurenka, 2003).

Another research study (Qu et al., 2021)investigated the potential therapeutic effects of cinnamaldehyde in rats with 2,4,6-trinitrobenzene sulfonic acid-induced (TNBS-induced) ulcerative colitis. The findings demonstrate that cinnamaldehyde possesses the ability to alleviate inflammatory injury by reducing the expression of IL6. Additionally, it inhibits NF-κB (Receptor for Advanced Glycation End-products) and TNF-α. Further examination unveiled that cinnamaldehyde notably decreases the levels of phosphorylated-Janus kinase 2 (p-JAK2) and p-signal transducer and activator of transcription 3 (p-STAT3). Simultaneously, it enhances the expression of the suppressor of cytokine signaling 3 (SOCS3) inhibitory protein.

Therefore, cinnamaldehyde inhibits TNBS-induced ulcerative colitis through anti-oxidant and anti-inflammatory properties, as well as by modulating the JAK2/STAT3/SOCS3 pathway. This evidence provides a good theoretical basis for the future use of cinnamaldehyde in the treatment of ulcerative colitis.

### 3.3 Suppression of periodontitis

Periodontitis is an oral inflammatory disease (Sudhakara et al., 2018), Ou et al. (Ou et al., 2022) investigated the effect of oral cinnamaldehyde on ligation-induced periodontitis in mice. They also examined the effect of cinnamaldehyde on mouse macrophages induced by *Porphyromonas gingivalis (P. gingivalis)* supernatant and human teeth. Regarding the impact on the inflammatory reaction of human periodontal ligament cells (HPDLC), mouse studies demonstrated that the oral intake of cinnamaldehyde effectively restrained bone erosion, the build-up of anaerobic bacteria, and the host’s immune-inflammatory response to ligature-induced periodontitis. *In vitro*, cinnamaldehyde inhibited the expression of IL6, IL8, TNF-α, and IL1β induced by (*P. gingivalis*) supernatant in RAW 264.7 cells and HPDLC. Moreover, cinnamaldehyde inhibited the expression of adherent cells and chemotaxis-related cytokines. These effects were accompanied by a decrease in the number of adherent HPDLC. These findings showed that cinnamaldehyde can improve H_2_O_2_-induced senescence of HPDLC, reduce the number of senescence-related β-galactosidase-positive cells, and reduce the expression of p53, p21, and p16.

In summary, oral administration of cinnamaldehyde can inhibit the progression of ligation-induced periodontitis in mice and significantly inhibit the symbiosis of microbial flora and host inflammatory response. These preliminary results confirm that cinnamaldehyde is an effective adjuvant treatment for the prevention of periodontitis.

### 3.4 Inhibition of psoriasis-like inflammation

Psoriasis is a skin disease with a multifaceted inflammatory autoimmune nature, impacting approximately 2%–3% of the population worldwide (Tseng et al., 2021). The most common causes of psoriasis are genetic, immune, and infectious factors (Girolomoni et al., 2017). For example, poor nutrition or a diet with inadequate intake of omega-3 fatty acids is linked to the development of psoriasis (Madden et al., 2020). Recent investigations have revealed that exposure to M5 triggers psoriasis-like alterations in cultured keratinocytes, encompassing enhanced cellular replication and inflammation, alongside compromised cellular differentiation (Nguyen et al., 2018). Consequently, enhancing the efficacy of pharmaceuticals for managing psoriasis constitutes the primary focus of contemporary investigations.

In the study conducted by Ding et al. (Ding et al., 2021), normal human epidermal keratinocytes (NHEK) were stimulated with M5 (IL1α, IL17A, IL22, tumor suppressor M, and TNF-α) to mimic the abnormal proliferation and differentiation of keratinocytes *in vitro*. In addition, cinnamaldehyde downregulated the expression of lipopolysaccharide in macrophages stimulated with pro-inflammatory cytokines TNF-α, IL1β, and IL6 (Kim et al., 2018). Cinnamaldehyde significantly inhibited cell proliferation and cell cycle progression, whereas it promoted the differentiation of M5-stimulated NHEK. It also significantly attenuated the extent of oxidative stress-related damage and ameliorated inflammatory damage induced by M5 in NHEK.

In summary, cinnamaldehyde protects NHEK against M5-induced proliferation and inflammatory damage by inhibiting the NF-κB and JNK signaling pathways. These results provide novel insights into the role of cinnamaldehyde in psoriasis. Therefore, cinnamaldehyde is also considered a potential drug for the treatment of psoriasis.

### 3.5 Relief of rheumatoid arthritis (RA)

RA is a heterogeneous, systemic, autoimmune disease (Gravallese and Firestein, 2023). Early diagnosis is key to successful treatment, particularly in patients with significant risk factors for poor outcomes. Although a therapeutic effect is observed in most patients, the currently available drugs do not cure the disease in a variety of patients. Therefore, there is an urgent need to develop new therapeutic drugs against RA.

RA is characterized by chronic persistent synovitis and various pathological features that result in structural damage, deformity, and loss of joint function. One of the main pathological features is synovial cell proliferation, specifically fibroblast-like Synoviocytes (FLS). These FLSs contribute to the inflammatory response in the joints and play a significant role in disease progression. In addition to FLS, other inflammatory cells (e.g., monocytes, polygonal B cells, and T cells) infiltrate the synovial tissue, further contributing to chronic inflammation. This inflammatory process leads to the erosion and destruction of bone tissue, exacerbating the structural damage in RA. Overall, the pathological features of RA ultimately lead to severe joint impairment and functional limitations (Bustamante et al., 2017). RA is a complex disease involving multiple types of cells. Among them, the overgrowth and redistribution of FLS in the synovial joint have been identified as significant pathological mechanisms contributing to RA. The abnormal proliferation and migration of FLS play a crucial role in the chronic inflammation and joint destruction observed in RA. Consequently, understanding the mechanisms underlying FLS overgrowth and redistribution is essential for developing effective treatments against RA. Through further investigation, advancements can be achieved in targeting and inhibiting FLS proliferation and migration, thereby alleviating the symptoms and halting the progression of RA (Wen et al., 2018). Prior research (Masoumi et al., 2021) has indicated notable alterations in the biological traits of FLS derived from RA. This is apparent in the varied morphology of cells, coupled with an escalation in cell proliferation and migration. Alongside the anomalous proliferation of FLS derived from RA, an impaired process of apoptosis in these cells may serve as an additional noteworthy mechanism in the context of RA (Wang et al., 2019). RA is a chronic inflammatory disease that affects the synovium, leading to synovial proliferation, synovitis, and cartilage erosion. RA-derived FLS plays a vital role in understanding the mechanisms involved in these pathological processes. By studying RA-derived FLS, researchers can gain important insights into the pathogenesis of RA. This understanding could pave the way for the development of novel therapeutic targets for the clinical treatment of this debilitating disease.

New research findings have indicated that the synovial membrane contains the phosphatidylinositol 3 kinase/protein kinase B (PI3K/AKT) signaling pathway. This specific pathway is of great importance in cellular signal transduction, as it plays a crucial role in various functions of FLS such as growth, proliferation, survival, apoptosis, adhesion, and migration. Furthermore, it exhibits close connections to the activation, adhesion, and angiogenesis of FLS derived from RA (Huang et al., 2019). Moreover, the PI3K/AKT pathway is implicated in leukocyte migration, phagocytosis, release of inflammatory factors, and activation of NF-κB in synovitis. It also holds significance in facilitating synovial neovascularization and degradation of stroll (Zhu et al., 2015).

XLi and Wang (Li and Wang, 2020)found that cinnamaldehyde significantly improved RA in type II collagen-induced rats. In another study, this effect was accompanied by a reduction of pro-inflammatory factors, proliferation, and metastasis. Cinnamaldehyde can reduce the expression levels of TNF-α, IL1β, and IL6 in RA-derived FLS. The results of Cell Counting Kit-8, 5-ethynyl-2′-deoxyuridine (EdU), and flow cytometry analyses showed that cinnamaldehyde significantly inhibited the proliferation of RA-derived FLS cells, reduced the number of EdU-positive cells, and promoted the apoptosis of RA-derived FLS cells. Further Western blotting experiments showed that cinnamaldehyde can simultaneously inhibit the activation of the PI3K/AKT signaling pathway in RA-derived FLS. The investigators also found that the PI3K/AKT signaling pathway activator 740Y-P can reverse the effect of cinnamaldehyde on the proliferation and metastasis of RA-derived FLS.

In conclusion, the study mentioned above has affirmed that the proliferation and metastasis of RA-derived FLS can be hindered by cinnamaldehyde through blockage of the PI3K/AKT signaling pathway. This discovery suggests that cinnamaldehyde offers therapeutic benefits for RA and presents a promising candidate for future treatment options.

### 3.6 Suppression of allergic rhinitis (AR)

AR is an inflammatory condition triggered by a response to the inhalation of an allergen, which is mediated by immunoglobulin E (IgE). The condition is characterized by sneezing, nasal congestion, and nasal itching (Wheatley and Togias, 2015). Inhaled allergens may reach the sinus mucosa and trigger an allergic reaction that leads to sinus mucosal congestion and impaired auxiliary clearance. AR can cause fatigue, headaches, cognitive impairment, and other symptoms that can reduce the quality of life.

Researchers (Hanci et al., 2016)employed a rodent model to assess the therapeutic efficacy of cinnamaldehyde in treating AR. According to the experimental observations, the allergic symptoms were most serious, the cilia were lost, and the inflammation was greater in the untreated group *versus* the treated group. The rate of allergy symptoms was significantly higher in the azelastine and cinnamaldehyde groups *versus* the control group. However, there was no significant difference in allergy symptoms between the azelastine and cinnamaldehyde groups. Histopathological analysis showed that the cinnamaldehyde group had vascular congestion and an increased number of goblet cells. Less plasma cell infiltration in the nasal mucosa of the rats was observed in the cinnamaldehyde group *versus* the control group. Analysis of the nasal mucosa showed less eosinophilic infiltration in the azelastine group compared with the control group. Thus, both azelastine hydrochloride and cinnamaldehyde can reduce allergy symptoms in AR model rats. Moreover, cinnamaldehyde can reduce vascular congestion and the infiltration of plasma cells, eosinophils, and inflammatory cells into the lamina propria. Therefore, cinnamaldehyde presents potential as an effective medication for addressing inflammation in rats suffering from AR.

## 4 Anti-microbial effect

Cinnamaldehyde was identified as the most active anti-microbial component in cinnamon, demonstrating anti-bacterial activity against *Salmonella S.)*, *E. coli*, *Streptococcus mutans (S. mutans)*, *A. hydrophila (Aeromonas hydrophila),* etc.

### 4.1 Salmonella

*Salmonella* (a common enteric pathogen) has attracted widespread attention worldwide. The inflammatory response caused by *Salmonella* infection is a crucial step in the pathogenic process (Galán, 2021). *Salmonella* is a foodborne pathogen that enters the intestinal tract after ingestion of contaminated food (Dong et al., 2019). *Salmonella* can disrupt the intestinal barrier function, leading to intestinal dysbiosis in the gastrointestinal tract. This disruption can have detrimental effects on the body, particularly in the liver. In cases of *Salmonella* infection, the interaction between the gut and the liver is triggered, resulting in liver injury. This injury is characterized by the infiltration of inflammatory cells, oxidative stress, hepatocyte apoptosis, and severe congestion. These manifestations indicate the severity of the liver damage caused by *Salmonella* infection (Shao et al., 2019).

Currently, *Salmonella* infections are mainly controlled through using antibiotics. Nevertheless, the excessive and prolonged usage of this specific treatment approach has led to the emergence of strains that are resistant to antibiotics. Therefore, it is necessary to identify new therapies for controlling *Salmonella* infections.

#### 4.1.1 Salmonella typhimurium (S. typhymurium)

*S. typhymurium* is a common foodborne pathogen that poses a serious risk to public health and food safety. *Salmonella typhimurium* type I hairs (T1F) are crucial for pathogenesis as they facilitate the adhesion of bacteria to mannose receptors on host cells and aid in their entry into these cells (Kalia et al., 2015). Elevated levels of serum alanine aminotransferase and aspartate aminotransferase have been found in mice infected with *S. typhymurium*. *Salmonella* hepatitis refers to a condition in which a patient with typhoid develops severe liver abnormalities and jaundice.

LYin et al. (Yin et al., 2022a)confirmed the inhibitory effect of cinnamaldehyde on T1F expression by hemagglutination inhibition assay, transmission electron microscopy, and biofilm assay. Proteomics techniques, PCR, and Western blotting were used to investigate the mechanism of action of cinnamaldehyde. The results showed that cinnamaldehyde could regulate the expression and transcription levels of fibrillin by regulating the expression of FimA, FimZ, FimY, FimH, and FimW. In addition, invasive assays demonstrated that cinnamaldehyde also reduced the cell adhesion ability of *S. typhymurium*. The results of animal experiments showed that cinnamaldehyde significantly reduced the levels of intestinal colonization and inflammatory cytokine expression, whereas it increased those of intestinal mucosal immune factors mucin 1 (MUC1) and MUC2. Thus, it was confirmed that cinnamaldehyde is a potential drug for targeting T1F in the treatment of *Salmonella* infection.

#### 4.1.2 *Salmonella* in chicken

LYin et al. (Yin et al., 2022b)investigated the protective effect of cinnamaldehyde on chicken liver cells challenged by *S*. Experiments revealed that cinnamaldehyde can significantly reduce the generation of ROS and malondialdehyde in liver cells, confirming its anti-oxidant effect. Cinnamaldehyde also inhibits the expression of pro-inflammatory cytokines and chemokines (e.g., IL1β, IL6, and TNF-α), thereby indicating its anti-inflammatory effect. In addition, cinnamaldehyde inhibited the apoptosis of hepatocytes. The findings from this study suggest that cinnamaldehyde has a protective impact on oxidative stress, inflammatory response, and apoptosis in the hepatic cells of chicks that are infected with *Salmonella gallinarum (S. gallinarum)*. By inhibiting the NF-κβ/CASP3 pathway, cinnamaldehyde can enhance the inflammatory response and decrease apoptosis induced by *S*. infection. Additionally, it exhibits anti-bacterial, anti-inflammatory, anti-oxidant, and anti-apoptotic properties in hepatocytes infected with *S. gallinarum*, rendering it a potential candidate for the development of drugs to treat *Salmonella* infection in poultry farming.

### 4.2 Effectiveness against pathogenic *E. coli*

*Escherichia coli* is a common bacterium in the human intestinal tract. However, some strains can cause serious diseases, ranging from urethritis to bloodstream infection (i.e., meningitis) through invasive routes (Manges et al., 2019). Traditional antibiotic therapy has failed to effectively treat patients with *E. coli* infection, and the overuse of antibiotics has led to adverse reactions and drug resistance (Arbab et al., 2022). Therefore, it is crucial to develop new treatment options for *E. coli* infection. As a natural product, cinnamaldehyde has been extensively studied and exhibited a broad-spectrum anti-bacterial effect (Liu et al., 2017). Therefore, investigation of cinnamaldehyde as a therapeutic drug against *E. coli* is of great importance.

WPereira et al. (Pereira et al., 2021)initially investigated the minimum inhibitory concentration (MIC) of cinnamaldehyde against *E. coli* using spectrophotometry; according to the results, the MIC was 1.2 ± 0.3 mM. Subsequently, the effects of cinnamaldehyde on the cell membrane structure and growth of *E. coli* were investigated using fluorescence microscopy, flow cytometry, and electron microscopy (He et al., 2018). Cinnamaldehyde was able to disrupt the integrity of the *E. coli* cell membrane, leading to intracellular material leakage and apoptosis. Moreover, cinnamaldehyde inhibited the growth of *E. coli*, and the duration of action was positively correlated with the concentration. In addition, mouse experiments were conducted to observe the effect of cinnamaldehyde on intestinal flora colonization. The findings indicated a significant reduction in the diversity of gut microorganisms in the cinnamaldehyde-treated group (Pereira et al., 2021). Moreover, cinnamaldehyde demonstrated noteworthy suppression of *E. coli* colonization and growth.

IFigueiredo et al. (Figueiredo et al., 2022) investigated the efficacy of cinnamaldehyde and its mechanism of action in mice. The animals received an intraperitoneal injection with *E. coli* and were classified into control, low-dose cinnamaldehyde, and high-dose cinnamaldehyde groups. The survival rate, pathological changes in the lungs and spleen, and immune-related indices of mice were determined. The investigators concluded that cinnamaldehyde can significantly improve the survival rate of mice infected with *E. coli*. The most pronounced treatment effect was noted in the high-dose group. The treatment could also reduce the pathological changes in the lungs and spleen of mice caused by *E. coli* infection. Cinnamaldehyde also enhanced the infiltration of inflammatory cells in the lungs and spleen of mice and significantly increased the serum concentrations of IL6 and TNF-α. Finally, cinnamaldehyde could reduce oxidative stress in mice by increasing the activity of superoxide dismutase (SOD) and glutathione peroxidase (GPX) (Liao et al., 2012). In conclusion, cinnamaldehyde can mitigate the effects of *E. coli* infection by enhancing the immune response and promoting redox homeostasis, thus improving the survival rate of mice.

A potential treatment for pathogenic *E. coli* infections lies in the use of cinnamaldehyde. This compound shows promising results and could be developed into a drug for treating such infections. Additionally, cinnamaldehyde offers novel insights and a theoretical foundation for the creation of natural fungicides or intestinal regulators.

### 4.3 S. mutans

*Streptococcus mutans* (Yu et al., 2022)is considered the most relevant bacterium for the transformation of non-pathogenic oral commensal flora into biofilm. In addition, it is one of the main pathogenic microorganisms that cause dental caries and periodontal disease. (Son et al., 2012). Conventional care modalities are often unable to eradicate this bacterium; hence, there is a need to identify a new compound for the treatment and prevention of this oral condition. Evaluating the potential impact of cinnamaldehyde on *S. mutans* holds both theoretical and practical importance due to its anti-bacterial properties.

ZHe et al. (He et al., 2019) investigated the anti-bacterial activity of cinnamaldehyde against *S. mutans* biofilm by measuring the MIC, minimum bactericidal concentration, and growth curve through various experimental methods (e.g., thin-layer plate method, fluorescence microscopy, and scanning electron microscopy). The crystal violet method and 3-(4,5-dimethylthiazol-2-yl)-2,5-diphenyltetrazolium bromide (MTT) method (He et al., 2019) were used to determine the effects of different concentrations of cinnamaldehyde and different culture durations on biofilm biomass and metabolism. The biofilms were visualized using confocal laser scanning microscopy, (He et al., 2019), and the surface hydrophobicity, aggregation, acid production, and acid resistance of bacterial cells treated with cinnamaldehyde were evaluated. The gene expression of virulence-related factors (glucosyltransferase B [GTFB], GTFC, GTFD, glucan binding protein B [GBPB], COMDE, VICR, CIAH, lactate dehydrogenase [LDH], and RelA) was detected by real-time PCR(He et al., 2019). The results showed that, at sub-MIC concentrations, cinnamaldehyde reduced biofilm biomass and metabolism. According to the images obtained through confocal laser scanning microscopy, the biofilm coverage area decreased as the concentration of cinnamaldehyde increased. Cinnamaldehyde increases the hydrophobicity of the cell surface, reduces the aggregation of *S. mutans*, and inhibits acid production and acid resistance. The presence of cinnamaldehyde downregulated gene expression in biofilms. Ali et al. also found that cinnamaldehyde inhibited biofilm formation in cocci such as *Enterococcus faecalis* and *Staphylococcus* spp. (Albano et al., 2019; Ali et al., 2021; Akshaya et al., 2023). This may be due to the inhibition of the gene coding for gelatinase in the biofilm by cinnamaldehyde, which corroborates the findings of the study by He et al. Thus, cinnamaldehyde suppresses microbial activity in *S. mutans* biofilms at the sub-MIC level by modulating the expression of genes associated with hydrophobicity, aggregation, acidogenesis, acid tolerance, and virulence.

In summary, experiments showed that cinnamaldehyde exerts a marked inhibitory effect on *S. mutans*, and can inhibit the growth of colonies and formation of biofilms. In addition, cinnamaldehyde can damage the integrity of the cell wall and membrane, and exert its anti-bacterial effect; thus, it demonstrates a certain inhibitory effect on *S. mutans*.

### 4.4 A. hydrophila

*Aeromonas hydrophila* (Igbinosa et al., 2012)is a typical Gram-negative short *bacillus* widely found in natural water, soil, fruits, vegetables, and other organisms. However, it is also one of the major pathogens causing human diseases, usually infecting humans through the oral, intestinal, and respiratory tracts. Infection causes severe gastroenteritis, septicemia, skin infections, and other symptoms. Antibiotics are currently the main drugs used to treat *A*. *hydrophila* infections (Stratev and Odeyemi, 2016). However, the drug-resistant strains of *A*. *hydrophila* exhibit a wide spectrum and high rate of drug resistance (Khor et al., 2018).

Based on this evidence, Chen et al. (Yin et al., 2020) investigated the anti-bacterial effects of cinnamaldehyde against *A*. *hydrophila*. The effects of cinnamaldehyde on cell growth, cell morphology, conductivity, LDH, protein metabolism, and DNA of *A*. *hydrophila* were examined using various methods, such as meat dip, fluorescence staining, and electron microscopy. (Yin et al., 2020). Following treatment with cinnamaldehyde, the conductivity, LDH activity, and DNA exocytosis of *A*. *hydrophila* were increased by 7.14%, 16.75%, and 20.29 μg/mL, respectively. Cinnamaldehyde could disrupt the cell membrane of *A*. *hydrophila*, thereby leading to cell death. The deformation and decrease in the number of *A*. *hydrophila* bacteria induced by cinnamaldehyde indicated a strong bactericidal effect. Further tests showed that the anti-bacterial mechanism of cinnamaldehyde against *A*. *hydrophila* was related to lipid peroxidation, DNA damage, and proteolysis of cell membranes.

In conclusion, cinnamaldehyde has exhibited strong *in vitro* activity for the inhibition of bacteria such as *A*. *hydrophila*. The inhibition was mainly achieved by disrupting the integrity of the cell structure, as well as interfering with DNA biosynthesis, protein metabolism, and cellular metabolism. This evidence demonstrates that cinnamaldehyde is a promising drug candidate for the treatment of infections with drug-resistant *A*. *hydrophila*.

## 5 Anti-tumor effects

Cinnamaldehyde has anti-proliferative and apoptotic activity against numerous types of human tumor cells, such as breast cancer, ovarian cancer, liver cancer, gastric cancer, non-small cell lung cancer (NSCLC), colorectal cancer (CRC), etc., (Banerjee and Banerjee, 2023).

### 5.1 Breast cancer

Breast cancer is among the most common types of cancer in women worldwide (Shafiee et al., 2022), and is associated with one of the highest mortality rates (Loibl et al., 2021). Traditional treatments, such as radiotherapy and chemotherapy, have demonstrated inconsistent efficacy and can be psychologically and physically burdensome. In recent years, researchers (Lu et al., 2010)have discovered that cinnamaldehyde exerts an inhibitory effect on tumor growth. However, the precise mechanism underlying this effect remains unknown, and further study is needed to identify specific targets.

YLiu et al. (Liu et al., 2020) investigated the mechanism and targets of cinnamaldehyde involved in the treatment of breast cancer. The results provided new ideas and directions for treatment and drug development in the future. Various methods were used to analyze the characteristics, mechanism of action, and targets of cinnamaldehyde (Liu et al., 2020). Firstly, cell experiments were used to study the inhibitory effect of cinnamaldehyde on different types of breast cancer. It was found that cinnamaldehyde could inhibit the proliferation, change the morphology, promote apoptosis, and reduce the invasiveness and migratory ability of MDA-MB-231 cells. Next, RNA sequencing technology was used to analyze the effect of cinnamaldehyde on transcription in breast cancer cells. The results showed that cinnamaldehyde regulates the expression of signaling pathways and proteins, including NF-κB, AKT, B-cell lymphoma 2 (BCL2), and BCL2-associated X (BAX). These signaling pathways and proteins play key roles in the initiation and progression of breast cancer. Cinnamaldehyde can affect these pathways and proteins, thereby inhibiting the growth and spread of breast cancer. Therefore, cinnamaldehyde has great medicinal value. The results of this study provide a valuable reference for the treatment of breast cancer and drug development.

### 5.2 Ovarian cancer

Ovarian cancer (Torre et al., 2018)is one of the most common malignant tumors in women. Despite significant advances in medical technology over the past few decades, ovarian cancer continues to be associated with limited therapeutic efficacy, high mortality rates, and changes in cellular epigenetics and signaling pathways leading to resistance to targeted therapies (Chandra et al., 2019). Therefore, the discovery of new drugs for the treatment of ovarian cancer has become an important research direction.

YWang et al. (Wang et al., 2022)used three groups of ovarian cancer cells, namely, SKOV3, A2780, and OVCAR-3, to produce a nude mouse subcutaneous xenograft tumor model. Wound healing, plate cloning, and Cell Counting Kit-8 assays were conducted to examine the effects of cinnamaldehyde on ovarian cancer cells. Transwell assays were used to assess cell proliferation and invasion, (Wang et al., 2018),while Western blotting and flow cytometry analyses were employed to measure the levels of apoptosis. The findings indicated that cinnamaldehyde, when cultured *in vitro*, can effectively hinder the viability of ovarian cancer cells. Moreover, the results obtained from wound healing and Transwell assays further demonstrated that cinnamaldehyde possesses inhibitory properties against the proliferation and invasion of A2780 and SKOV3 cells.

In addition, cinnamaldehyde can also promote apoptosis by increasing the expression of poly (ADP-ribose) polymerase (PARP) and CASP3 in ovarian cancer cells (Wang et al., 2022). A mechanistic study revealed that cinnamaldehyde inhibited the PI3K/AKT signaling pathway induced by epidermal growth factor (EGF), and decreased the phosphorylation levels of the mechanistic target of rapamycin kinase (MTOR), PI3K, and AKT. Cinnamaldehyde can eliminate the epithelial–mesenchymal transition process induced by EGF and AKT-specific activator SC79. In addition, cinnamaldehyde in the body can significantly inhibit the progression of ovarian cancer (Hong et al., 2016). Moreover, it can inhibit the activation of the EGF signaling pathway and has a significant inhibitory effect on EGF-induced epithelial–mesenchymal transition.

The study yielded significant findings regarding the potential of cinnamaldehyde in controlling and preventing ovarian cancer. The results revealed that cinnamaldehyde possesses the ability to impede the advancement and spread of ovarian cancer cells in laboratory settings and living organisms. Importantly, these findings highlight the potential of cinnamaldehyde as an effective agent for inhibiting the growth and progression of ovarian cancer. This discovery provides a new idea and research basis for the development of treatment plans in the future.

### 5.3 Hepatocellular carcinoma

Cinnamaldehyde also showed obvious anti-proliferative activity in human liver cancer HepG2 cells. The anti-proliferative activity was determined using the 2,3-bis(2-methoxy-4-nitro-5-sulphophenyl)-2H-tetrazolium-5-carboxanilide (XTT) method, and the expression of apoptosis-related proteins was detected by Western blotting. The experiments revealed that cinnamaldehyde induces the activation of p53 and CD95 (APO-1) signaling pathways to inhibit the proliferation and apoptosis of liver cancer HepG2 cells (Ng and Wu, 2011). It can also jointly control transcription factors by activating the extracellular signal-regulated kinase 1/2 (ERK1/2), AKT, and JNK signaling pathways. The nuclear translocation and transcriptional activity of nuclear factor E2-related factor 2 (NRF2) allows it to bind to the enhanced subsequence of the anti-oxidant response element. This leads to upregulation of the expression of biphasic detoxification enzymes and stimulates glutathione production (Huang et al., 2011b).

Lin et al. (Lin et al., 2013) used human liver cancer cell lines as an experimental model, induced cell apoptosis by different concentrations of cinnamaldehyde, and explored the effects by Western blotting, flow cytometry, and cytochrome C release experiments. (Lin et al., 2013). The results showed that cinnamaldehyde promoted the apoptosis of liver cancer cells by activating the mitochondrial death pathway. Specifically, cinnamaldehyde upregulated BAX and p53 expression, whereas it downregulated BCL2 and cellular inhibitor of apoptosis 2 (CIAP2) expression. These effects resulted in decreased mitochondrial membrane potential, release of intramitochondrial enzymes, and activation of CASP9 and CASP3. In addition, the data showed that cinnamaldehyde-induced apoptosis of liver cancer cells could be suppressed by cyclosporine A (a mitochondrial calcium-regulated protein kinase inhibitor) and benzyloxycarbonyl-Val-Ala-Asp-fluoromethyl ketone (Z-VAD-fmk; a broad-spectrum cysteine ​​protease inhibitor) inhibition. This evidence demonstrated the importance of mitochondrial calcium-regulated protein kinases and cysteine proteases in this process.

In summary, through the in-depth study of the mechanism of cinnamaldehyde-induced apoptosis of liver cancer cells, the mentioned evidence provides an important theoretical basis for the future use of cinnamaldehyde in the treatment of liver cancer.

### 5.4 Gastric cancer

Gastric cancer is characterized by high incidence (Wu et al., 2023) and mortality rates worldwide (Smyth et al., 2020). Despite several advances achieved in the past decades regarding the prevention and treatment of gastric cancer, there is a lack of effective treatments. The current treatment for gastric cancer mainly involves traditional modalities, such as surgery, chemotherapy, and radiotherapy (Takahashi et al., 2013). Nevertheless, these treatment options are characterized by limitations and are linked to side effects. Thus, there is a need to develop effective and safe therapeutic drugs for gastric cancer.

To investigate the mechanism of action of cinnamaldehyde in gastric cancer cells, studies (Kim, 2021) have used various experimental methods, including MTT and clone formation assays, Western blotting and immunofluorescence staining analyses, flow cytometry, and gene silencing techniques. The investigators used cinnamaldehyde to treat gastric cancer cells and examined its effect on promoting apoptosis. Cinnamaldehyde significantly reduced cell activity and induced gastric cancer cell death. Western blotting analysis revealed that treatment with cinnamaldehyde inhibited the expression of anti-apoptotic proteins, such as BCL2, BCL-extra-large (BCL-xL), and myeloid cell leukemia 1 (MCL1), whereas it increased that of apoptotic proteins, such as BAX and BCL2 associated agonist of cell death (Pattingre et al., 2005). These findings indicated that cinnamaldehyde induced gastric cancer cell death by inhibiting the expression of anti-apoptotic proteins. Further experiments revealed that cinnamaldehyde may affect cellular gene transcription levels by altering the DNA methylation status. Cinnamaldehyde mediates endoplasmic network stress and cellular autophagic death through the PRKR-like endoplasmic reticulum kinase/C/EBP homologous protein (PERK/CHOP) signaling pathway, inhibition of G9a binding to beclin 1 (BECN1) and light chain 3B (LC3B) promoters, and dissociation of BCL2/BECN1 in gastric cancer cells. Specifically, the levels of G9a, a H3K9 methyltransferase, can be regulated by cinnamaldehyde. This regulation induces cell death in gastric cancer and activates the autophagic pathway. Cinnamaldehyde also induced the expression of autophagy-associated proteins LC3B and BECN1, and promoted the activity of pheromone splitting enzyme autophagy-related 3 (ATG3), thereby accelerating autophagy-mediated cell death.

The results of this study expanded the prospects of cinnamaldehyde application in the field of cancer and provided new ideas for the study of epigenetic modifications related to cancer therapy. Investigation of the biological mechanisms of cinnamaldehyde may lead to the identification of potentially useful anti-cancer therapeutic strategies.

### 5.5 Non-small cell lung cancer (NSCLC)

Lung cancer is the prevailing form of cancer and the foremost contributor to cancer-induced fatality. It is also estimated that the rate of lung cancer occurrence will increase (Chen et al., 2016). Approximately 85% of patients with lung cancer have NSCLC. NSCLC is one of the most common types of cancer worldwide (Lin et al., 2023), characterized by high morbidity and mortality rates. Over the past few decades, remarkable progress has been achieved in the treatment of lung cancer. Nevertheless, treatment effectiveness against NSCLC remains suboptimal. Traditional chemotherapy drugs have limited therapeutic efficacy, and drug resistance to these agents often occurs. Thus, researchers seek new drugs to overcome the above challenges. (Singh et al., 2021).

In recent years, studies have shown that cinnamaldehyde has anti-tumor effects. Ru Chen et al. (Chen et al., 2021) utilized whole-transcriptome sequencing to examine the impact of cinnamaldehyde on gene expression at the mRNA, miRNA, and long noncoding RNA levels. By constructing a competing endogenous RNA (ceRNA) network, the researchers identified several key molecules involved, including 13 mRNAs, six miRNAs, and 11 long noncoding RNAs. Further analysis revealed that three of these mRNAs (SOCS1, BTG2, and BTK) are associated with the JAK/STAT signaling pathway, which is involved in cancer development. Additionally, the NF-κB signaling pathway has been linked to cancer and influenced by cinnamaldehyde. Notably, the study demonstrated that SOCS1, BTG2, and BTK play significant roles in the anti-NSCLC activity of cinnamaldehyde. Further investigations (Nagano et al., 2016; Fu et al., 2017; Yan et al., 2017) identified several noncoding RNAs (LINC01504, LINC01783, THUMPD3-AS1, has-miR-155-5p, has-miR425-5p, and has-miR-7-5p) that mediate the inhibition of the malignant phenotype of NSCLC by cinnamaldehyde. Moreover, the JAK/STAT signaling, RNA degradation, and NF-κB signaling pathways were determined as key regulatory pathways involved in the effects of cinnamaldehyde on NSCLC.

In summary, cinnamaldehyde can block proliferation, induce apoptosis, and inhibit the migratory ability and invasiveness of NSCLC cells.

### 5.6 Colorectal cancer (CRC)

CRC refers to a type of malignant tumor of the digestive tract with a high incidence worldwide (Hu et al., 2022). It is highly invasive and metastatic, posing a great threat to the lives of patients (Siegel et al., 2023). Early symptoms of CRC are often unnoticed. At the time of diagnosis, CRC is often at an advanced stage and may have metastasized to the liver. The occurrence, development, and metastasis of various types of tumors are closely associated with the PI3K/AKT signaling pathway, which plays a crucial role in intracellular signal transduction. This pathway is indispensable for several cellular processes, including cell metabolism, apoptosis, survival, differentiation, and proliferation. Consequently, the complex process of CRC carcinogenesis necessitates cumulative alterations in multiple genes and pathways (Suman et al., 2013). Notably, the expression levels of AKT and p-AKT are significantly higher in CRC tissues *versus* normal tissues (Dardor et al., 2023). This leads to malignant transformation of cells, as well as tumor cell migration, adhesion, and degradation of the extracellular matrix. Therefore, the PI3K/Akt pathway is considered a potential target for cancer therapy.

Li et al. (2015) investigated the impact of cinnamaldehyde on the molecular and cellular levels in human colon cancer cells. Differentiated CRC cell types (SW480, HCT116, and LoVo) with varying degrees of invasiveness were exposed to cinnamaldehyde at concentrations of 20, 40, and 80 μg/mL for 24 h, alongside a control group. The results showed that cinnamaldehyde effectively hindered the growth of human CRC cells in a time- and dose-dependent manner. Furthermore, cell invasion and adhesion were significantly suppressed based on the results obtained from Transwell and cell-matrix adhesion assays. The expression of E-cadherin was upregulated, whereas that of matrix metallopeptidase 2 (MMP2) and MMP9 was downregulated by cinnamaldehyde. Additionally, an increase in apoptosis rate was observed. The expression of pro-apoptotic genes was enhanced, whereas that of anti-apoptotic genes was decreased, further confirming the pro-apoptotic impact of cinnamaldehyde. This is also supported by Zhang et al. and Wu et al. who found that cinnamaldehyde significantly inhibited the growth of all types of colon cancer cells and induced apoptosis of colon cancer cells by blocking the G0/G1 phase (Wu et al., 2019; Zhang et al., 2023). Furthermore, the mechanism underlying cinnamaldehyde-induced apoptosis was explored by modulating the PI3K/AKT pathway using insulin-like growth factor 1 (IGF1) and PI3K inhibitor (LY294002). The study found that cinnamaldehyde significantly suppressed the transcriptional activity of PI3K/AKT, while IGF1 exhibited anti-apoptotic activity. Thus, it can be concluded that cinnamaldehyde triggers apoptosis in CRC cells by inhibiting the activation of the PI3K/AKT pathway. Moreover, it inhibits CRC cell invasion and adhesion and counteracts the activation of the PI3K/AKT signaling pathway induced by IGF1. Zhang et al. also investigated the effect of cinnamaldehyde on the PI3K-Akt pathway, and their results instructed that the target of cinnamaldehyde’s action was HSPD1, and that cinnamaldehyde and HSPD1 co-localized and affected the PI3K-Akt pathway in HCT-116 cells. (Zhang et al., 2023).

These findings provide an experimental basis for the use of cinnamaldehyde as a drug for colon cancer. However, additional basic studies are needed to verify the anti-tumor activity of cinnamaldehyde.

## 6 Treatment of diabetes

The incidence rate of diabetes, a chronic disease characterized by increased glucose concentration in the blood, is increasing at an alarming rate (Zhang and Ren, 2015). The diabetic population is mainly affected by vascular diseases involving multiple organ systems. These can be divided into microvascular and macrovascular complications that can lead to nephropathy or cardiovascular disease, increase the risk of myocardial infarction, stroke, and amputation, and lead to premature death (Gray and Jandeleit-Dahm, 2014). The mechanisms of hyperglycemia-induced vascular disease are complex and associated with endothelial dysfunction, oxidative stress, and glycosylation of numerous vascular proteins.

It has been shown that cinnamaldehyde lowers glycolipids in diabetic animals by increasing glucose uptake and insulin sensitivity in adipose and skeletal muscle tissue. These effects improve hepatic glycogen synthesis, restore islet dysfunction, reduce the gastric emptying rate, and improve diabetic kidney and brain disease. Cinnamaldehyde exerts the above effects through multiple signaling pathways, including peroxisome proliferator-activated receptors (PPARs), AMP-activated protein kinase (AMPK), PI3K/insulin receptor substrate 1 (PI3K/IRS1), retinol-binding protein 4/glucose transporters type 4 (RBP4/GLUT4), ERK/JNK/p38/mitogen-activated protein kinase (ERK/JNK/p38/MAPK), transient receptor potential cation channel subfamily A member 1/ghrelin (TRPA1/ghrelin), and NRF2 signaling pathways (Zhu et al., 2017).

### 6.1 Hypoglycemic effect

GLUT4 on the membrane of skeletal muscle cells can improve the utilization of sugar in skeletal muscle and relieve hyperglycemia. Cinnamaldehyde can reduce the levels of blood sugar by upregulating the expression of the GLUT4 gene in mouse skeletal muscle (Nikzamir et al., 2014). It was found that the overexpression of protein tyrosine phosphatase 1B (PTP1B) in tissue cells reduces the activity of protein tyrosine kinase. Consequently, insulin receptors cannot bind insulin, thereby causing insulin resistance and eventually leading to the development of type 2 diabetes. Cinnamaldehyde has demonstrated inhibitory activity against PTP1B, thus helping to treat or prevent type 2 diabetes and obesity (Saifudin et al., 2012). Other studies have shown that cinnamaldehyde can also enhance the anti-oxidant defense against ROS generated under hyperglycemic conditions, thereby preventing the loss of islet β cells and exerting a hypoglycemic effect (Yuan et al., 2011). In addition, cinnamaldehyde inhibits the biological effects of diabetic nephropathy induced by advanced glycation end products. This is achieved by inhibiting the JAK2/STAT1/STAT3 cascade or the activated nitric oxide (NO) pathway (Huang et al., 2015).

### 6.2 Reduction of body fat

By diminishing the accumulation of visceral fat deposition, cinnamaldehyde exhibits its proficient potential in enhancing diabetic adipose tissue. Consequently, it stimulates lipolysis and thermogenesis of fatty acid oxidation.

Tamura et al. (Tamura et al., 2012) examined C57BL/6 mice fed a high-fat/high-sugar diet for 1 month. The results showed that the addition of cinnamaldehyde (0.1%–1.0%) to the high-fat/high-sugar diet reduced the weight of mesenteric adipose tissue in mice, and tended to reduce the weight of perirenal and epididymal adipose tissue.

The potential effects of cinnamaldehyde on diabetes may partially derive from its role in regulating the metabolism of adipose tissue by stimulating the breakdown of fats and the oxidation of fatty acids. Cinnamaldehyde can increase the levels of hormone-sensitive lipase (HSL) in 3T3-L1 preadipocytes while suppressing the levels of cyclopropane and glycerol-3-phosphate dehydrogenase. Additionally, it can also lower the activation of peroxisome proliferators in adipocytes, and inhibit the expression of the receptor (PPARγ) and the CCAAT/enhancer-binding protein-α (CEBP-α) genes (Huang et al., 2011a). In mice fed a high-fat diet, cinnamaldehyde increased the expression of target genes fatty acid synthase (FAS), sterol regulatory element binding protein 1 (SREBP1), stearyl-coenzyme desaturase 1 (SCD1), adipocyte fatty acid binding protein αP2, p-AMPK, and p-acetyl-CoA carboxylase (p-ACAC). Moreover, it inhibited 3-phosphoglycerol acyltransferase activity in epididymal adipose tissue and 3T3-L1 adipocytes. In addition, treatment with cinnamaldehyde (10 mg/kg) increased the expression of lipolytic genes (HSL, patatin phospholipase domain 2 [PNPLA2], and monoglyceride lipase [MGL]) in visceral adipose tissue of mice fed a high-fat diet (Khare et al., 2016).

### 6.3 Metabolism of substances in muscle tissue by cinnamaldehyde

Muscle tissue plays a crucial role in glucose metabolism, as it is the primary insulin target tissue responsible for consuming >85% of the glucose in the body. In individuals with diabetes, insufficient production or resistance to insulin can result in GLUT4 translocation impairment in skeletal muscle. This ultimately leads to a reduction in glucose uptake by the muscle tissue.

Cinnamaldehyde can improve glucose metabolism in skeletal muscle tissue. Anand et al. (Anand et al., 2010) treated streptozotocin-induced rats with cinnamaldehyde for 2 months to restore the levels of GLUT4 and increase those of glycogen in skeletal muscle. In addition, Gannon et al. (Gannon et al., 2015) showed that cinnamaldehyde activates the PPARγ coactivator 1α (PGC1α) and its downstream effector myocyte enhancer factor 2 (MEF2), and increases the expression of GLUT4 in C2C12 cells. It was concluded that the PGC1α/MEF2/GLUT4 pathway in C2C12 cells regulates mitochondrial metabolism and, thus, plays an anti-diabetic role.

### 6.4 Effect of cinnamaldehyde on hepatic glycogen

Diabetes may lead to a decrease in the levels of hepatic glycogen synthase, thereby affecting glycogen storage and synthesis (Hesselink et al., 2016). Pyruvate kinase (PK) and phosphine pyruvate carboxykinase (PEPCK) are two major enzymes involved in glycogen synthesis by regulating glycolysis and gluconeogenesis (Zhao et al., 2016). Insulin deficiency has been significantly associated with elevated PEPCK and decreased PK activity (Medagama, 2015). Cinnamaldehyde has positive effects on the diabetic liver by regulating PK and PEPCK activity, reducing the levels of retinol-binding protein 4 (RBP4), enhancing glycogen synthesis, and improving glucose metabolism and insulin sensitivity. Kumar et al. (Kumar et al., 2012) reported that treatment of diabetic rats with cinnamaldehyde for 28 days could significantly increase the concentration of glycogen in the liver. Moreover, the administration of cinnamaldehyde to streptozotocin-induced diabetic rats for 2 months caused a surge in hepatic PK and PEPCK activities, as well as mRNA levels. The findings revealed that cinnamaldehyde potentially enhances glycogen synthesis and suppresses gluconeogenesis, thereby contributing to its hypoglycemic properties.

Diabetes can lead to increased levels of various enzymes in the plasma, such as aspartate aminotransferase, alanine aminotransferase, LDH, alkaline phosphatase, and liver acid phosphatase. However, treatment of diabetic rats with cinnamaldehyde has been found to normalize the activities of these enzymes. Additionally, RBP4, which is primarily produced in the liver and adipose tissue, tends to increase in the serum and liver in response to high glucose levels (Mahfouz et al., 2016). Excessive elevation of RBP4 further hampers the signaling of insulin and enhances hepatic utilization of glucose. Administration of cinnamaldehyde can substantially diminish the concentrations of RBP4 in both serum and the liver, suggesting its potential to ameliorate hepatic function in individuals with diabetes.

## 7 Therapeutic effects on cardiovascular diseases

Epidemiological studies have shown that the morbidity and mortality rates of cardiovascular diseases are increasing annually, posing a great threat to the social economy and human health worldwide. In addition, the incidence rates of these diseases are increasing rapidly, particularly in developing countries (Sabatine et al., 2017). Cinnamaldehyde can be used in the treatment of cardiovascular diseases. Cardiovascular diseases, such as atherosclerosis, cardiac hypertrophy, etc., are the main cause of death in humans. Therefore, the identification of new effective targets and therapeutic approaches may provide a theoretical basis for the treatment of cardiovascular diseases (Shang et al., 2021).

### 7.1 Improvement of atherosclerosis

Atherosclerosis seriously endangers the health and quality of life of individuals. The pathogenesis of arteriosclerosis includes damage to the vessel wall by mechanical injury, viral infection, and other pathogenic factors, such as infection and dyslipidemia (particularly oxidized LDL abnormality), which induce a severe chronic inflammatory response (MY et al., 2008). The oxidative stress response and inflammatory response transition of vascular endothelial cells are important factors in the progression of atherosclerosis (Mei et al., 2008). Cinnamaldehyde is a widely distributed natural product, suitable for use in beverages, medicines, perfumes, cosmetics, etc. It has been shown that cinnamaldehyde exerts therapeutic effects against high-fat-induced atherosclerosis through its hypolipidemic, antioxidant, and anti-inflammatory activities (Ito et al., 2019).

Basma S. (Ismail et al., 2022). Ismail et al., examined atherosclerosis induced by a high-fat diet for 10 weeks in experimental rats. Continuous administration of cinnamaldehyde via gavage demonstrated a significant reduction in elevated levels of total cholesterol, triglycerides, LDL-cholesterol, very low-density lipoprotein-cholesterol, and free fatty acids in rats. Moreover, it exhibited a substantial increase in the reduced levels of high-density lipoprotein-cholesterol. Cinnamaldehyde effectively mitigated the heightened cardiovascular risk index (Shang et al., 2021), while simultaneously improving the diminished anti-atherosclerotic index (Das et al., 2022). Additionally, it reduced the activities of serum creatine kinase, creatine kinase-MB, LDH, and aspartate aminotransferase activities. Furthermore, cinnamaldehyde exhibited enhancement in cardiac anti-oxidant activity by eliciting a decrease in malondialdehyde levels and an increase in glutathione S-transferase, SOD, catalase, reduced glutathione, and GPX activities. In addition, cinnamaldehyde downregulated the mRNA expression levels of IL1β, IL6, IL17, and TNF-α. Therefore, this compound successfully exerted a therapeutic effect against high-fat diet-induced atherosclerosis through its hypolipidemic, antioxidant, and anti-inflammatory properties.

Li et al. (Liu et al., 2019), investigated the protective effect and potential mechanism of cinnamaldehyde against atherosclerosis using a high-fat diet-fed apolipoprotein E^−/−^ (ApoE^−/−^) mouse model of atherosclerosis. It was found that the levels of serum LDL-cholesterol, triglycerides, and total cholesterol were increased, whereas those of high-density lipoprotein-cholesterol were decreased, in ApoE^−/−^ mice fed a high-fat diet. Treatment with cinnamaldehyde significantly reduced the overproduction of inflammatory cytokines (TNF-α, IL6, NO, and monocyte chemoattractant protein-1 [MCP-1]) and lipid levels. Moreover, cinnamaldehyde increased the levels of high-density lipoprotein-cholesterol and downregulated the activity of lipid peroxidation product malondialdehyde in serum. Furthermore, it reduced the area of atherosclerotic plaque in ApoE^−/−^ mice. Cinnamaldehyde also decreased the expression of MMP2 and attenuated the hyperphosphorylation of IκBα and p65 NF-κB. Thus, the investigators concluded that cinnamaldehyde may achieve its anti-atherosclerotic effects through the IκB/NF-κB signaling pathway.

### 7.2 Inhibition of cardiac hypertrophy

Yang et al. (Yang et al., 2015)used the aortic band method to induce myocardial hypertrophy in mice. Premixed cinnamaldehyde in the diet was administered to the mice 1 week after aortic banding. Echocardiography and catheter hemodynamic parameters were measured at week 7. The degree of myocardial hypertrophy was evaluated by pathological and molecular analyses of mouse heart specimens. The effects of cinnamaldehyde on myocardial hypertrophy, fibrosis, and dysfunction caused by aortic banding were observed. The parameters investigated in this study included heart weight/body weight, lung weight/body weight, heart weight/tibia length, echocardiography and hemodynamic parameters, histology, and gene expression of hypertrophy and fibrosis markers. It was found that cinnamaldehyde intervention improved systolic and diastolic abnormalities, and reduced cardiac fibrosis in mice. Further studies showed that cinnamaldehyde can block the ERK signaling pathway that is significantly activated by pressure overload, thus providing new insights into the molecular mechanism of pathological cardiac hypertrophy.

### 7.3 Inhibition of platelet activation and agglutination

Research has demonstrated that cellular senescence and programmed cell death have a crucial regulatory function in maintaining the internal environment stability of the body. Endothelial cells play an important role in various cardiovascular and cerebrovascular diseases (Libby, 2021). Studies have found that cinnamaldehyde can decrease the levels of nicotinamide adenine dinucleotide phosphate (NADPH) enzyme, reduce ROS, change the mitochondrial membrane potential, reduce caspase pathway conduction, and promote the reduction of apoptotic cells by activating the NRF2 pathway (Wang et al., 2015). The toll-like receptor 4/NF-кB (TLR4/NF-кB) pathway can increase the ratio of BAX/BCL2 activated by CASP9/CASP3, while the endothelial nitric oxide synthase/NO (eNOS/NO) pathway can downregulate PARP. Hence, the TLR4/NF-кB induced through the inhibition of β and eNOS/NO increase by cinnamaldehyde will further accelerate the apoptosis of endothelial cells (Yang et al., 2021). Cinnamaldehyde alleviated heart failure induced by lipopolysaccharide in a mouse model. The mechanism underlying this effect may be related to blockage of the TLR4/NADPH oxidase 4 (TLR4/NOX4) and MAPK/NF-кB pathways in the oxidative stress response induced by lipopolysaccharide. Previous studies have found that cinnamaldehyde can inhibit the activation of BAX and CASP3, increase the levels of BCL2, and reduce the heart damage caused by ischemia/hypoxia. Cinnamaldehyde promotes apoptosis by regulating WNT/beta-catenin (WNT/CTNNB), PERK/CHOP, AMPK/MTOR, and other signal transduction pathways (Huang et al., 2020).

In summary, the mentioned studies highlighted the therapeutic effects of cinnamaldehyde, thereby providing a scientific basis for the use of this compound in clinical practice to prevent and treat cardiovascular diseases.

## 8 Other pharmacological effects

The main functions of kidneys are to excrete waste, regulate water and electrolyte balance, and maintain acid-base balance. Nevertheless, aging is associated with a reduction in renal function due to multiple factors (Denic et al., 2016). It has been reported that autophagy plays an important role in this process (Lenoir et al., 2016). Brustolin et al. (Brustolin et al., 2022) have found that cinnamaldehyde possesses anti-leishmanial activities. In addition, cinnamaldehyde has anti-depressant properties (Ying et al., 2015). (Table 1).

**TABLE 1:** Pharmacological effects of cinnamaldehyde.

Pharmacological effects	Adopting the model		Adopting the model	Main mechanisms	Main targets	References
Anti-Inflammatory	Gastritis caused by *H*. *pylori*		①*H. pylori*-induced AGS cell model	① Inhibition of NF-κB activation and downregulation of Helicobacter pylori-induced IL8 expression in AGS cells	NF-κB	Jibran Sualeh Muhammad (Jibran et al., 2015)
	Ulcerative colitis		①2,4,6-trinitrobenzenesulfonic acid (TNBS)-induced ulcerative colitis in rats	①Reduces inflammatory damage by reducing interleukin-6, inhibiting nuclear factor-κB and tumor necrosis factor-α expression through antioxidant and anti-inflammatory properties and modulating JAK2/STAT3/SOCS3 pathway	JAk2/STAT3/SOCS3	ZhijieLu (Shu-Lan et al., 2021)
	Periodontitis		①Pg supernatant-induced RAW 264.7 and HPDLCs model	①Inhibition of Pg supernatant-induced expression of IL6, IL8, TNFA, IL1B in RAW 264.7 and HPDLCs	①P53, P21 and P16	Jiang Chen (YanJing et al., 2022)
			②Ligated periodontitis mice	②Inhibit the expression of adherent cells and chemotaxis-related cytokines to reduce adherent HPDLCs		
				③Improvement of H2O2-induced cellular senescence in HPDLCs, reduction in the number of senescence-associated -β-galactosidase-positive cells, and reduction in the expression of P53, P21, and P16		
	Psoriasis-like inflammation		①M5 (IL-1α, IL-17A, IL-22, tumor suppressor M and TNF-α) stimulated normal human epidermal keratinocytes (NHEKs)	①Downregulation of lipopolysaccharide expression levels	①Phosphorylation p) inhibitors of NF - κB, p - p65 and p - JNK	Zhenzhen Ding (Zhenzhen et al., 2021)
				②Significantly inhibited proliferation and cell cycle progression and promoted differentiation of M5-stimulated NHEKs		
				③Significantly attenuated the extent of oxidative stress-induced damage in M5-stimulated NHEKs and improved M5-induced inflammatory damage in NHEKs		
				④Significantly downregulated the expression levels of NF-κB, p-p65 and p-JNK phosphorylation p) inhibitors in M5-stimulated NHEKs		
	Rheumatoid arthritis		①Type II collagen-induced in rats	①Blocking PI3K/AKT signaling pathway inhibits proliferation and metastasis of RA-FLS	①PI3k/AKT	Xiang Li (Xiang and Yue, 2020)
	Allergic rhinitis		①Dissolve 0.3 mg of ovalbumin (OVA) in 1 mL of saline and prepare sensitization solution by using 30 mg of aluminum hydroxide (40 mg/mL) as adjuvant for intraperitoneal injection into rats	①Reduces vascular congestion and reduces infiltration of plasma cells, Eosinophils, and inflammatory cells into the lamina propria		Deniz Hancı (Deniz et al., 2016)
Antibacterial	*S*	*S*. *typhimurium*	①*S. typhimurium* infection	①Regulate the expression and transcript levels of FIMA, FIMZ, FIMY, FIMH and FIMW	①FIMA, FIMZ, FIMY, FIMH and FIMW	Lizi Yin(Lizi et al., 2022b)
				②reduce the cell adhesion ability of *S. typhimurium*		
				③Significantly reduce the expression levels of intestinal colonization and inflammatory cytokines, and increase the levels of intestinal mucosal immune factors MUC1 and MUC2		
		*S*. in chickens	①Chicks infected with *S*	①Reducing reactive oxygen species (ROS) and malondialdehyde (MDA) production in hepatocytes	①NF-Kβ/caspase-3	Lizi Yin(Lizi et al., 2022a)
				②Inhibit the expression of pro-inflammatory cytokines and chemokines		
				③Inhibit hepatocyte apoptosis		
	Pathogenic *E. coli*		①Mice were injected intraperitoneally with *E. coli*	①Disrupt the integrity of *E. coli* cell membrane, leading to intracellular material leakage and apoptosis	①Superoxide dismutase (SOD) and glutathione peroxidase (GPx)	Isabella F. S. Figueiredo (IFS et al., 2022)
				②Inhibit the growth of *E. coli*		
				③Significantly inhibit the colonization and proliferation of *E. coli*		
				④Enhanced inflammatory cell infiltration in the lung and spleen, and significantly increased serum concentrations of IL-6 and TNF-α		
				⑤Increased superoxide dismutase (SOD) and glutathione peroxidase (GPx) activities to reduce oxidative stress		
	*S. mutans*		① *S. mutans*	①Regulation of hydrophobicity, aggregation, acid production, acid resistance, and virulence gene expression, and inhibition of microbial activity in *S. mutans* biofilms		Zhiyan He (Zhiyan et al., 2019)
	*A. hydrophila*		1 *A*. *hydrophila*	①Disrupt the integrity of cell structure		Jiehao Chen (Lizi et al., 2020)
				②Interfere with DNA biosynthesis and protein metabolism as well as cellular metabolism		
Anti-tumor	Breast Cancer		①MDA-MB-231 cells	①Inhibit MDA-MB-231 cell proliferation, alter MDA-MB-231 cell morphology, promote MDA-MB-231 cell apoptosis, and reduce MDA-MB-231 cell invasion and migration ability	①NF-κ, AKT, BCL-2, BAX	Bowen Yu(Yufei et al., 2020)
				②Regulate the expression of signaling pathways and proteins, including NF-κB, AKT, BCL-2, BAX		
	Ovarian Cancer		①Ovarian cancer cells	①Inhibit the viability of ovarian cancer cells	①PI3K/Akt	Yue Wang (Zhong et al., 2022)
			②A2780 and SKOV3 cells	②Inhibit the proliferation and invasion ability of A2780 and SKOV3 cells	②EGF	
				③Increase the expression of PARP and Caspase3 in ovarian cancer cells and promote apoptosis		
				④Inhibit EGF-induced PI3K/Akt signaling pathway and decrease the phosphorylation level of mTOR, PI3K, and Akt		
				⑤Inhibit the activation of the EGF (epidermal growth factor) signaling pathway and suppress EGF-induced epithelial mesenchymal transition (EMT)		
	Liver Cancer		①Hepatocellular carcinoma cells	①Induced activation of p53 and CD95 (APO-1) signaling pathways to inhibit the proliferation and apoptosis of hepatocellular carcinoma HepG2 cells	①p53 and CD95 (APO-1)	Iin LT (Liang-Tzung et al., 2013)
				②Activation of ERK1/2, Akt and JNK signaling pathways jointly control the nuclear translocation and transcriptional activity of transcription factor Nrf2, which binds to the subsequence of enhanced antioxidant response element to upregulate the expression of diphasic detoxification enzymes and stimulate glutathione production	②ERK1/2, Akt and JNK	
				③Causes upregulation of Bax and p53 expression and downregulation of Bcl-2 and CIAP2 expression, leading to a decrease in mitochondrial membrane potential, release of intra-mitochondrial enzymes and activation of caspase 9 and caspase 3	③Mitochondrial death pathway	
	Stomach Cancer		①Stomach cancer cells	①Inhibit the expression of cellular anti-apoptotic proteins and induce gastric cancer cell death	①Anti-apoptotic proteins such as Bcl-2, Bcl-xL and Mcl-1, and apoptotic proteins such as Bax and Bad	Tae Woo Kim(TW, 2022)
				②Alter DNA methylation status and affect cellular gene transcription levels	②PERK-CHOP	
				③Inhibit the binding of G9a to Beclin-1 and LC3B promoters and the dissociation of Bcl-2-Beclin-1 in GC cells through PERK-CHOP signaling pathway, mediating endoplasmic network stress and cellular autophagic death	③H3K9 methyltransferase G9a	
				④Regulation of H3K9 methyltransferase G9a levels leads to cell death in gastric cancer cells and further directs activation of the autophagic pathway	④Autophagy-related proteins LC3B and Beclin-1	
				⑤Induce the expression of autophagy-related proteins LC3B and Beclin-1 and promote the activity of pheromone splitting enzyme Atg3, thereby accelerating autophagy-mediated cell death		
	Non-small cell lung cancer		①Non-Small Cell Lung Cancer cells	①Inhibits the proliferation of non-small cell lung cancer cells, induces apoptosis, and inhibits the migration and invasion of non-small cell lung cancer cells	①JAK/STAT	Ru Chen(Ru et al., 2020)
					②NF-κB	
	Colorectal Cancer		①Colon cancer cells	①Inhibit the ability of rectal cancer cells to increase in value, invasion and adhesion	①PI3K/Akt	Jiepin Li(Jeipin et al., 2016)
				②Promote apoptosis of rectal cancer cells		
Other	Diabetes		①STZ often induced diabetes in rats and mice model	①Glucose-lowering effect	①PTP-1B, JAK2-STAT1-STAT3 cascade or activated NO pathway	Jau-Shyang Huang (Jau-Shyang et al., 2015)
			①Mice with high fat and high sugar	①Lower body fat	①Hormone-sensitive lipase (HSL); fatty acid synthase (FAS), sterol regulatory element binding protein 1 (SREBP1), stearoyl coenzyme desaturase 1 (SCD-1) and adipocyte fatty acid binding protein αP2; pro-lipolytic genes (HSL, Patatin phospholipase structural domain 2 (PNPLA2) and monoglyceride lipase (MGL)	Bo Huang
			①STZ-induced diabetic rats	①Improving the grape of muscle tissue Glucose metabolism	③PGC-1α/MEF2/GLUT4 in C2C12 cells	
			①STZ-induced diabetic rats	①Improve glucose metabolism and insulin sensitivity in the liver	④PK and PEPCK	
	Cardiovascular disease		①Rats on oral high-fat diet	①Improve atherosclerosis	①IκB/NF-κB	
			①Myocardial hypertrophy was induced in mice using the aortic banding (AB) method	①lnhibits the cardiac hypertrophy	①ERK	
			①Lipopolysaccharide-induced heart failure model in mice	①Inhibition of platelet activation and coagulation	①TLR4/NOX4 and MAPKs/NF-к B pathways; Wnt/beta-catenin, PERK/CHOP, AMPK/mTOR and other signaling pathways	
	Kidney disease		①D-galactose (D-gal) construction for rat aging model	①Promotes autophagy in kidney cells and tissues	①miR-155	
					②PI3K/Akt	

Abbreviations: AKT, protein kinase B; ATG3, autophagy related 3; BAD, BCL2 associated agonist of cell death; BAX, BCL2 associated X; BCL2, B-cell lymphoma 2; BCL-xL, BCL-extra-large; BECN1, beclin 1; CASP3/9, caspase 3/9; CHOP, C/EBP, homologous protein; CIAP2, cellular inhibitor of apoptosis 2; CTNNB, beta catenin; *E. coli*, *Escherichia coli*; EGF, epidermal growth factor; ERK1/2, extracellular signal-regulated kinase 1/2; FAS, fatty acid synthase; GLUT4, glucose transporters type 4; GPX, glutathione peroxidase; HPDLC, human periodontal ligament cells; IκB, inhibitor of NF-κB; IL1α/6/8/17A/22, interleukin 1α/6/8/17A/22; JAK2, Janus kinase 2; JNK, JUN N-terminal kinase; LC3B, light chain 3B; MCL1, myeloid cell leukemia 1; MEF2, myocyte enhancer factor 2; MGL, monoglyceride lipase; MTOR, mechanistic target of rapamycin kinase; MUC1, mucin 1; NF-κB, nuclear factor-kappa B; NO, nitric oxide; NOX4, NADPH, oxidase 4; NRF2, nuclear factor E2-related factor 2; PARP, poly(ADP-ribose) polymerase; PEPCK, phosphine pyruvate carboxykinase; PERK, PRKR-like endoplasmic reticulum kinase; PGC1α, PPARγ, coactivator 1α; PI3K, phosphatidylinositol 3 kinase; PK, pyruvate kinase; PNPLA2, patatin phospholipase structural domain 2; PTP1B, protein tyrosine phosphatase 1B; SOCS3, suppressor of cytokine signaling 3; STAT3, signal transducer and activator of transcription 3; STZ, streptozotocin; TLR4, toll like receptor 4; TNBS, 2,4,6-trinitrobenzenesulfonic acid; TNF-α, tumor necrosis factor alpha.oxide; NOX4, NADPH, oxidase 4; NRF2, nuclear factor E2-related factor 2; PARP, poly(ADP-ribose) polymerase; PEPCK, phosphine pyruvate carboxykinase; PERK, PRKR-like endoplasmic reticulum kinase; PGC1α, PPARγ, coactivator 1α; PI3K, phosphatidylinositol 3 kinase; PK, pyruvate kinase; PNPLA2, patatin phospholipase structural domain 2; PTP1B, protein tyrosine phosphatase 1B; SOCS3, suppressor of cytokine signaling 3; STAT3, signal transducer and activator of transcription 3; STZ, streptozotocin; TLR4, toll like receptor 4; TNBS, 2,4,6-trinitrobenzenesulfonic acid; TNF-α, tumor necrosis factor alpha.

## 9 Signal pathway involved in the main action of cinnamaldehyde

At the molecular level, cinnamaldehyde is involved in the regulation of various signaling pathways to exert its anti-inflammatory and anti-tumor effects, with key signaling pathways playing different important roles.

### 9.1 Effect of cinnamaldehyde on the PI3K/AKT signal pathway

**FIGURE 2:**
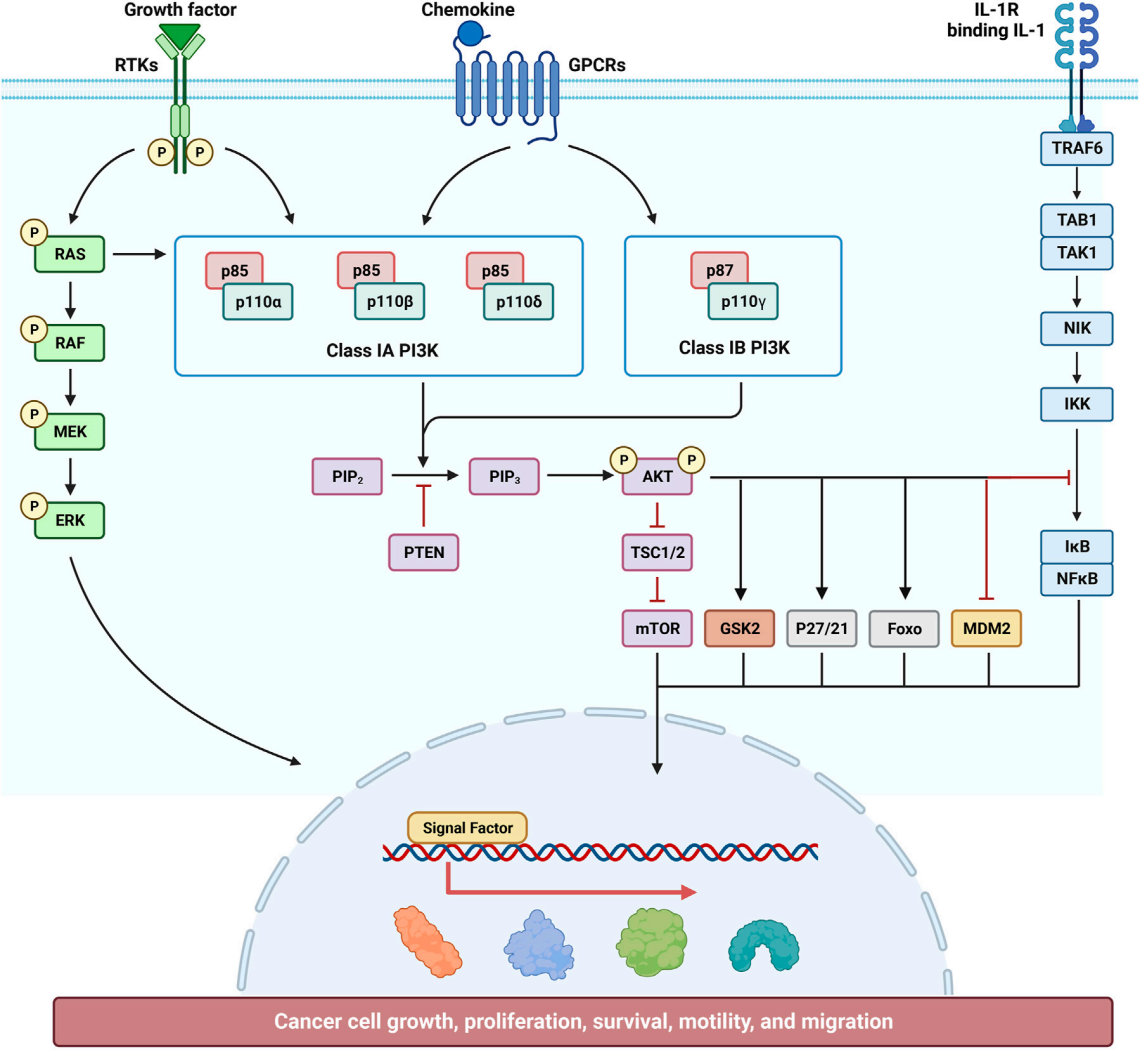
Effect of cinnamaldehyde on the phosphatidylinositol 3 kinase/protein kinase B (PI3K/AKT) signaling pathway.

Cinnamaldehyde can inhibit RA by inhibiting the activation of the PI3K/AKT signaling pathway in the synovial membrane. Research revealed that cinnamaldehyde inhibited the EGF-induced PI3K/AKT signaling pathway and, thus, had a certain inhibitory effect on ovarian cancer cells. Further analysis using kidney cells showed that cinnamaldehyde can also regulate the PI3K/AKT signaling pathway to further promote autophagy, thus achieving a protective effect on the kidneys (Figure 2)

### 9.2 Effect of cinnamaldehyde on the NF-κB signal pathway

**FIGURE 3:**
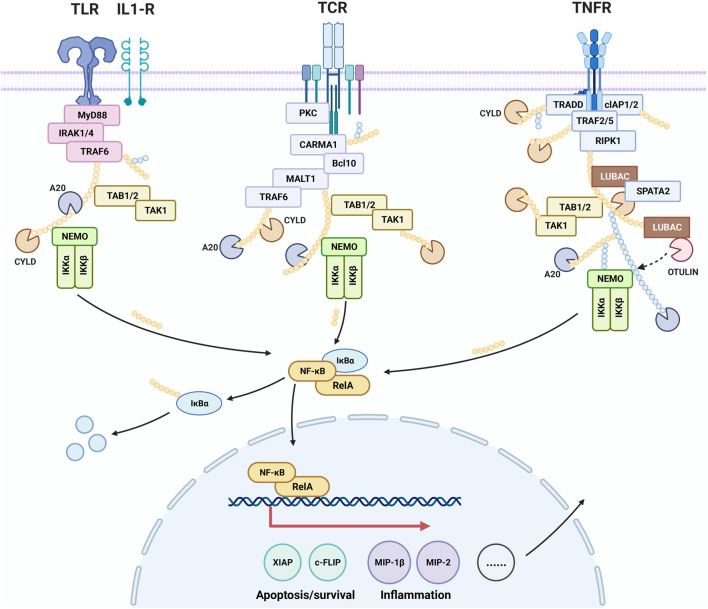
Effect of cinnamaldehyde on the nuclear factor-kappa B (NF-κB) signaling pathway.

NF-κB contributes to the control of cell survival, differentiation, and proliferation. Activation of NF-κB is associated with a variety of diseases including cancer, autoimmune diseases, neurodegenerative diseases, and cardiovascular diseases. It has been found that cinnamaldehyde exerts a therapeutic effect on psoriasis-like inflammation by inhibiting the NF-κB pathway. Moreover, it improves gastritis caused by *H. pylori* by inhibiting the activation of NF-κB in AGS cells. Analysis of the inhibitory effect of cinnamaldehyde on tumor growth demonstrated that blockage of the NF-κB pathway led to inhibition of the growth and spread of breast cancer cells, resulting in an anti-cancer effect. (Figure 3).

## 10 Summary

Cinnamaldehyde, exhibits a broad pharmacological spectrum, influencing diverse diseases through intricate mechanisms, targeting a wide array of diseases from inflammation to bacterial infections, cancer, diabetes, cardiovascular disease, etc. Its anti-inflammatory effects are rooted in inhibiting the NF-κB pathway and reducing pro-inflammatory mediators like interleukins and TNF-α, beneficial for treating gastritis, ulcerative colitis, and rheumatoid arthritis. For example, in *H. pylori*-induced gastritis, cinnamaldehyde suppresses NF-κB activation and IL8 expression in AGS cells. In antimicrobial action, cinnamaldehyde disrupts *Salmonella* and *E. coli* by affecting pilin levels and cell membrane integrity, curbing bacterial biofilms. It exhibits notable efficacy against various cancers, including breast, ovarian, and colorectal, by inducing apoptosis and hindering cell cycle progression. Its molecular targets include NF-κB, AKT, and caspase pathways. Cinnamaldehyde also shines in metabolic regulation, enhancing glucose uptake and insulin sensitivity, crucial for managing diabetes and obesity. Its cardiovascular benefits are seen in atherosclerosis and cardiac hypertrophy mitigation, influenced by lipid-lowering, antioxidant, and anti-inflammatory effects. Furthermore, it shows potential in kidney disease treatment by promoting autophagy through pathways like miR-155 and PI3K/Akt. This compound’s vast therapeutic promise necessitates further research to exploit its full potential in clinical applications, focusing on its detailed mechanisms of action and safety profiles.

Despite the considerable therapeutic promise of cinnamaldehyde, its clinical application is constrained by the intricate nature of the biochemical pathways it affects, including NF-κB and AKT. The complexity of these pathways can result in inconsistent therapeutic effects and unintended interactions, especially in conditions characterized by the dysregulation of multiple signaling pathways. To overcome these obstacles, future research should prioritize the development of selective pathway modulators that specifically target critical components within these pathways, potentially boosting both the effectiveness and safety of cinnamaldehyde. Additionally, the pharmacokinetics of cinnamaldehyde, encompassing its absorption, distribution, metabolism, and excretion (ADME), remain inadequately characterized. This deficiency impedes the refinement of dosage protocols and the forecasting of potential interactions with other drugs. Employing sophisticated analytical techniques for enhanced pharmacokinetic profiling is crucial to deepen our understanding of these characteristics, which would support the formulation of more precise dosing strategies and enhance therapeutic outcomes.

By addressing these scientific and clinical challenges and directing research towards these sophisticated areas, we can unlock the full therapeutic capacity of cinnamaldehyde, paving the way for more efficacious and safer therapeutic interventions.
